# Health Profiles in Developmental Age: An Analysis of the Eating Habits and Lifestyles of a Sample of Italian Children

**DOI:** 10.3390/children12101296

**Published:** 2025-09-25

**Authors:** Bianca Maria Bocci, Dario Lipari, Ilaria Manini, Andrea Pammolli, Rita Simi, Antonella Miserendino, Elena Frongillo, Mattia Fattorini, Cinzia Massini, Daniele Rosadini, Riccardo Frazzetta, Giacomo Lazzeri

**Affiliations:** 1 Post Graduate School of Public Health, University of Siena, 53100 Siena, Italy; d.lipari@student.unisi.it (D.L.); manini3@unisi.it (I.M.); a.miserendino2@student.unisi.it (A.M.); e.frongillo@student.unisi.it (E.F.); giacomo.lazzeri@unisi.it (G.L.); 2 Department of Molecular and Developmental Medicine, University of Siena, 53100 Siena, Italy; pammolli2@unisi.it (A.P.); rita.simi@unisi.it (R.S.); 3 Research Center on Health Prevention and Promotion (CREPS), University of Siena, 53100 Siena, Italy; 4 Interuniversity Research Centre on Influenza and Other Transmissible Infections (CIRI-IT), 16132 Genoa, Italy; 5 Public Hygiene and Nutrition Unit (IPN), Amiata Senese, Val d’Orcia and Valdichiana Senese Area, Azienda USL Toscana Sud-Est, 53100 Siena, Italy; mattia.fattorini@uslsudest.toscana.it (M.F.); cinzia.massini@uslsudest.toscana.it (C.M.); daniele.rosadini@uslsudest.toscana.it (D.R.); riccardo.frazzetta@uslsudest.toscana.it (R.F.)

**Keywords:** nutritional surveillance, eating habits, lifestyle, children, health education, physical activity, fruit and vegetable consumption, sugary drinks, BMI, socioeconomic status

## Abstract

**Background:** The adoption of a healthy lifestyle and eating habits in children represents a major public health objective worldwide, with significant implications for the development of chronic non-communicable diseases in adulthood. In Italy, the “OKkio alla SALUTE” Surveillance System (National Institute of Health) has been in place since 2007 to periodically monitor the nutritional status and health-related behaviors of children aged 8 to 9 years old. **Methods:** Data were collected as part of the 2023 nutritional surveillance survey in the Tuscany Region through questionnaires completed by both children and their parents. A cluster sample design was adopted. The weight and height of children were directly measured. Logistic regression analysis was used to examine the association between measured variables (unhealthy eating habits and lifestyles) and overweight or obesity. **Results:** A total of 1427 children participated. In our sample, 17% of children were overweight, 5.7% were obese, and 1.3% were severely obese, totaling 24% of children classified as overweight. Tuscany’s rates are lower than the national average of 28.8%. Children whose parents had a low level of education were nearly twice as likely to consume sugary drinks daily (OR_adj = 1.97; 95% CI: 1.22–3.18) and to lead a sedentary lifestyle (OR_adj = 1.99; 95% CI: 1.33–2.97). Children from families reporting financial hardship were more likely to consume fruit and vegetables less than once a day (OR_adj = 2.35; 95% CI: 1.12–4.92) and to spend more time in sedentary activities (OR_adj = 3.30; 95% CI: 1.66–6.56). Regarding overweight, including obesity, children from economically challenged families had nearly double the risk of being overweight compared to those from financially stable households (OR_adj = 1.81; 95% CI: 1.09–2.98). **Conclusions**: The aim of our study was to evaluate which family factors are associated with unhealthy lifestyles in order to assess and, if appropriate, confirm the need for targeted and integrated interventions involving families, schools, and local communities to promote healthy lifestyles and effectively combat childhood obesity in Tuscany.

## 1. Introduction

Adopting healthy eating habits and lifestyles from early childhood is a key preventive measure against the development of non-communicable diseases, which today represent a major public health issue [1,2]. Inadequate nutrition during childhood and adolescence, crucial stages for overall development, can compromise growth and increase the risk of chronic diseases. An adequate intake of fruits and vegetables is essential for children’s physical and mental development and decreases the risk of cardiovascular diseases, cancer, and insulin resistance in adulthood [3,4,5].

Numerous studies confirm that unhealthy eating habits, such as the consumption of sugary drinks [6,7,8,9], skipping or having an inadequate breakfast [10,11,12], and the frequent consumption of snacks and fast food [13,14,15,16,17,18], are associated with an increased risk of overweight and obesity.

In recent years, children’s health has become a social problem in most countries worldwide. According to the latest reports from the World Health Organization (WHO) and the Joint Research Centre of the European Commission, one of the main concerns is the growing prevalence of risky behaviors associated with poor eating habits, physical inactivity, and excess weight. Specifically, a high consumption of sugary drinks, low intake of fruits and vegetables, sedentary behavior, and prolonged screen time (TV, tablets, smartphones) have become widespread practices among children [19].

Data collected in the sixth Childhood Obesity Surveillance Initiative (COSI) of the WHO Regional Office for Europe survey, conducted between 2022 and 2024, involving 37 countries and approximately 470,000 children, shows that childhood overweight and obesity remain major public health challenges. Twenty-five percent of children aged 7 to 9 are overweight (including obesity) and ten percent are obese. There continue to be significant differences between countries, with an overall prevalence of overweight ranging from 9% to 42% and obesity from 3% to 20%. In particular, the highest prevalence of overweight and obesity in children was found in southern European Countries such as Cyprus, Greece and Italy [20].

In Italy, the “OKkio alla SALUTE” cross-sectional national survey, part of the COSI, showed that 3 out of 10 children in 2023 were overweight (19% overweight, 7.3% obese and 2.6% severely obese), with a higher prevalence of overweight among girls than boys (19.8% vs. 18.3%) and a higher prevalence of obesity among boys than girls (10.3% vs. 9.4%). As regards overweight, from 2008 to 2023, Italy has seen a statistically significant decrease in overweight children, from 23.2% in 2008 to 19% in 2023, while obesity has remained essentially stable (from 12% in 2008 to 9.8% in 2023) [21].

Tuscany’s demographics, as in Italy, show a continuous trend towards an aging population, with a low birth rate and a geographical distribution concentrated mainly in the central and north-western areas [22]. The seventh survey by “OKkio alla SALUTE” showed slight improvements in the prevalence of overweight, down compared to previous surveys, while obesity remained stable [21].

The aim of our study was to identify the family factors associated with unhealthy lifestyles in children to confirm the need for targeted intervention involving multiple stakeholders, including families, schools and local communities, to promote healthy lifestyles and combat childhood obesity in Tuscany.

## 2. Materials and Methods

This analysis is based on data collected in Tuscany as part of the “OKkio alla SALUTE” national surveillance initiative, coordinated by the Italian National Institute of Health (ISS) [21]. The study population consisted of third-grade primary school pupils, mostly aged 8 to 9 years, who were considered capable of providing reliable responses to the items in a structured questionnaire.

With the support of the Ministry of Education, lists of public and private schools and classes with the number of children in each class were obtained from regional school authorities. A stratified cluster sample design was used, following the WHO cluster survey methodology [23], with classes set as the sampling unit. All children of the selected classes were invited to participate. The sample size was estimated in such a way as to achieve a precision of 3% in the estimate of BMI (the most important outcome variable) for regional estimates and of 5% for Local Health Unit (LHU) estimates [21,23].

Data collection was conducted using a standardized methodology and four validated questionnaires: one administered to children during school hours and another completed by parents at home. The parental questionnaire covered several areas, including the child’s physical activity levels, sedentary behavior, dietary habits, and parental perceptions of the child’s weight status.

In addition, trained personnel collected anthropometric data, specifically weight and height, using standardized measurement protocols. The prevalence of overweight and obesity was determined by calculating Body Mass Index (BMI), defined as body weight in kilograms divided by height in meters squared.

Data was collected between March and June 2023. Participants were excluded from the study if they were absent on the day of data collection or if informed consent from a parent or legal guardian was not obtained. Further methodological details are available in previously published sources [24,25]. Children were classified as overweight or obese based on their BMI, calculated as weight in kilograms divided by height in meters squared, according to age- and sex-specific cut-off values established by the World Obesity Federation–International Obesity Task Force (WOF-IOTF) [26].

A series of dietary behaviors were selected as variables, including the consumption of (1) fruit and/or vegetables and (2) sugary drinks. Parents were asked how frequently their children consumed these items during a week. Response options included never, less than once a week, a few days a week (1–3 days), almost every day (4–6 days), once a day, and two or more times per day (with separate categories for 2–3 times and 4 or more times per day for fruit and vegetables). For the purposes of this analysis, dietary intake was dichotomized as “at least once a day” versus “less than once a day.”

Regarding physical activity, children were classified as “active” if either the child or their parents reported that the child had played outdoors and/or participated in physical activity or sports on the day before the survey.

Sedentary behaviors were assessed by measuring the time spent watching television and/or playing video games, as well as using computers, tablets, and mobile phones on a typical school day [27]. Information was obtained from the parents’ questionnaire and categorized as ≤2 h and >2 h, as internationally recommended [27].

Socioeconomic characteristics included in the analysis were gender (male or female), area of residence (population ≤10,000; 10,001–50,000; >50,000 in a non-metropolitan area; or metropolitan/peri-metropolitan area), parents’ citizenship (both parents Italian; one foreign parent; or both parents foreign), parents’ level of education (classified as low = less than high school diploma; medium = high school diploma; high = university degree or higher), and perceived economic difficulties (based on how easily the family manages day-to-day expenses, with response options very easily, fairly easily, with some difficulty, or with great difficulty).

Frequency distributions of children and prevalence rates of outcome variables were calculated based on socioeconomic characteristics. In cases of missing data, percentages were computed using only available (non-missing) cases. Prevalence was stratified by socioeconomic characteristics, and differences between groups were assessed using Pearson’s χ^2^ test. Logistic regression models were used to explore associations between explanatory variables and each outcome variable, with results presented as mutually adjusted odds ratios (ORs) and 95% confidence intervals (CIs). Statistical analyses were performed using SPSS, version 26.

## 3. Results

A sample of 1708 children were enrolled across 84 schools. Only 2% of schools declined to participate. In addition, 142 parents (8.3%) did not consent to their child’s participation, and 123 children (7.2%) were absent. Nine children were excluded due to severe physical or mental disabilities and/or specific nutritional needs, while four children (0.2%) could not be measured and were therefore excluded. Finally, 19 children fell outside the target age range (<8 or >9 years) and were not included in the analysis. In total, data from 1427 children aged 8/9 years who were present and completed the questionnaire were analyzed. Table 1 presents the main characteristics of the participating children. The gender distribution was approximately equal (49.4% male and 50.6% female). Most of the children (58.9%) were 8 years old. Regarding parental citizenship, 74.9% of the children had two Italian parents, followed by 18.3% with two foreign parents. As for the area of residence, the distribution was relatively balanced across the three categories, 10,000–50,000 inhabitants, >50,000 (non-metropolitan area), and metropolitan/peri-metropolitan area, with each comprising roughly 30% of the sample. Fewer than 10% of the children resided in areas with populations under 10,000.

Overall, 40% of the children had at least one parent with a high level of education, while 44.9% had at least one parent with a medium level of education. A total of 15.3% of children had both parents with a low level of education. Nearly 38% of children lived in families that reported experiencing financial difficulties.

Table 2 presents the outcome variables stratified by gender. No statistically significant differences were observed. However, nearly 25% of the sample consumed fruit and/or vegetables less than once per day (22.9% of boys vs. 20.9% of girls; *p* > 0.05). Regarding sugary drink consumption, 18.0% of boys and 18.8% of girls reported daily intake (*p* > 0.05). In terms of physical activity, over 80% of children were not active on the day prior to the survey. Additionally, 40.9% of the sample (43.2% of boys vs. 38.4% of girls; *p* > 0.05) reported spending more than two hours per day watching television or using electronic devices such as video games, tablets, computers, or mobile phones. The prevalence of overweight (including obesity) was 22.6% among boys and 25.5% among girls. However, the prevalence of obesity alone appeared to be slightly higher in boys than in girls (7.6% vs. 6.5%).

Table 3 illustrates the prevalence of outcome variables based on socioeconomic characteristics. The percentage of children with “less healthy” behaviors was higher among children whose parents had a lower level of education and perceived greater economic hardship.

Children whose parents had a lower level of education showed a higher prevalence of overweight and obesity. Specifically, 30.8% of children with less educated parents were overweight or obese, compared to 19.7% among those with highly educated parents. The difference was also evident when considering obesity alone, with a prevalence of 10.4% in the lowest education group versus 4.2% in the highest. Dietary behaviors followed a similar trend. Daily consumption of fruit and vegetables was less frequent among children whose parents had a lower educational level (24.6%) than among those with medium (23.9%) or high (18.2%) education levels (*p* < 0.001). Conversely, daily intake of sugary drinks was more common among children in the low education group (30.6%) compared to those in the medium (18.6%) and high (12.1%) education groups (*p* < 0.001). Moreover, sedentary behavior was significantly more prevalent among children with less educated parents (54.4%; *p* < 0.001). A comparable pattern emerged when stratifying by perceived family economic status, with less favorable behavioral and health outcomes among children from financially disadvantaged households. No significant differences in overweight including obesity, obesity, or physical inactivity were found by parental citizenship. However, children with two foreign parents reported a higher daily consumption of both fruit/vegetables and sugary drinks compared to children with two Italian parents. Screen time was also greater: 50.7% of children with two foreign parents spent more than two hours per day using electronic devices, compared to 38.4% among those with Italian parents. No statistically significant associations were observed in relation to area of residence.

Multivariate logistic regression models (Table 4) confirmed most of the associations identified in the unadjusted analyses. Gender and residential areas were not associated with unhealthy behaviors. Parental education emerged as a key determinant: children whose parents had a low level of education were nearly twice as likely to consume sugary drinks daily (OR_adj = 1.97; 95% CI: 1.22–3.18) and to lead a sedentary lifestyle (OR_adj = 1.99; 95% CI: 1.33–2.97), after adjusting for all other covariates. Economic difficulties also had a significant impact: children from families reporting financial hardship were more likely to consume fruit and vegetables less than once a day (OR_adj = 2.35; 95% CI: 1.12–4.92) and to spend more time in sedentary activities (OR_adj = 3.30; 95% CI: 1.66–6.56). Additionally, children with two foreign parents were at higher risk of consuming sugary drinks daily (OR_adj = 2.89; 95% CI: 1.98–4.22). Regarding overweight including obesity, children from economically challenged families had nearly double the risk of being overweight compared to those from financially stable households (OR_adj = 1.81; 95% CI: 1.09–2.98).

## 4. Discussion

Our study, based on data from a representative regional sample, shows that a high percentage of children in Tuscany still do not follow recommendations on nutrition, physical activity and sedentary behavior, and that the prevalence of overweight is high. The data for Tuscany show a relatively better situation than in other Italian regions, but it is still far from optimal European standards [28]. Socio-economic inequalities, environmental influences and lack of physical activity at school are key factors. Low socioeconomic status is clearly associated with high rates of obesity and greater recourse to less healthy lifestyles, such as less physical activity and more time spent in sedentary activities (television, video games) [25].

Although a slight change in the prevalence of overweight children was found, decreasing compared to previous waves of our survey collections, the prevalence of overweight/obese children remained stable (2010 28.5%, 2012 25.8%, 2014 26.8%, 2016 27%, 2019 25.8%, 2023 24%) [21,24]. Compared with data of the “OKkio alla SALUTE” Italian survey, Tuscany has slightly lower overweight and obesity values (24% vs. 28.8%). The prevalence of overweight and obesity in Italy follows a geographical trend, with lower values in the north, 15.3% in Bolzano (12% overweight and 3.3% obese), and higher values in the south, with 43.2% (24.6% overweight and 18.6% obese) in Campania [21]. Among the children in the Tuscany region, 1.3% are severely obese, 5.7% are obese, 17% are overweight, 74.2% are normal weight and 1.8% are underweight. Therefore, 24% of children are overweight, which includes both overweight and obesity [21]. A correlation has also emerged between the presence of an overweight or obese parent in the family and the presence of cases of overweight and obesity in childhood [29].

Previous studies have shown, in line with our findings, that parents do not have an accurate picture of their child’s weight status [30,31]. In families with overweight and obese children, perception does not change based on the child’s sex but increases with the mother’s level of education [32]. Finally, the type of delivery was significantly associated with the child’s weight, while we found no significant association with breastfeeding, as shown in other studies [33,34].

Previous studies have shown an association between being overweight and the habit of skipping breakfast [35,36,37,38]. In Tuscany, only 58.4% of children have an adequate breakfast, and 32.6% do not have one, with a higher prevalence in girls of 34.8% compared to boys, 30.2%. This attitude, as has also emerged from other international studies [39,40,41], is higher in children with mothers who have a lower educational qualification, of primary or lower secondary school. In Tuscany, only 36.2% of the children consume an adequate mid-morning snack, while 63.3% consume an inadequate one. According to the guidelines for healthy and correct nutrition, five portions of fruit and/or vegetables should be consumed during the day [42]. As for fruit and vegetable consumption, only 22.7% and 14.6% of children consume them 2–3 times a day, respectively. In addition, 51.6% of families have included fruit as a snack in their daily diet and 66% have increased their consumption of vegetables. Research shows that there is a higher prevalence of children who do not eat breakfast and do not consume fruit and vegetables frequently in households where the mother’s level of education is lower, in line with the results of other international studies [43,44]. Among unhealthy eating habits, 6.5% of children consume sugary drinks almost every day, while the consumption of savory and sweet snacks “several times a day, every day” is 0.8% and 4.9%, respectively. In addition, 42.5% of children never consume legumes or consume them less than once a week. No differences were found based on the sex of the children.

The 2023 survey data showed that children do not engage in much physical activity. On the day before the survey, 12.1% of children were inactive, a higher percentage for those living in non-metropolitan areas with more than 50,000 inhabitants, while only 16.6% participated in a physical activity program at school. These results are in line with other Italian studies [45,46]. Parent questionnaires show that about 4 out of 10 children (43.1%) participate in at least one hour of structured sports activities 2 days a week, 15.1% do not participate in any sports activities, and only 2.6% participate in sports activities 5 to 7 days a week. Boys participate in structured sports activities on more days than girls. In addition, it emerged that approximately 2 out of 10 children (22.2%) play outdoors 2 days a week, 7% do not play even 1 day, and only 32.2% play 5 to 7 days a week. On the morning of the survey, 24.9% of children said they walked, cycled, rode a scooter or skated to school. Regarding walking or cycling to school, a statistically significant difference was found between different types of residential areas. Within the group of children who do not engage in physical activity, 69.1% of mothers believe that their child gets enough physical activity and 6.4% believe that their child gets a lot of physical activity [47,48]. Regarding sedentary activities, parents reported that on school days, 59.1% of children watch TV or use video games/tablets/mobile phones for less than 2 h per day, while 35.3% watch TV or use video games/tablets/mobile phones for 3–4 h a day and 5.6% for at least 5 h, with slightly higher values at the weekend. Exposure is more frequent among boys (43.2% versus 38.8%) [49,50]. Finally, it emerged that 38.2% of children have a television in their bedroom, as do their peers living in other countries. Considering television viewing as a potential influencing factor may be important, and recommendations to limit viewing time may be useful for the development of healthy lifestyles [51].

Maintaining a balanced diet and a healthy body weight from childhood onwards is essential for protecting health in adulthood and old age. The deficiencies highlighted by our analyses are a wake-up call, as they could herald an increase in public health problems in the coming decades. The data collected in our study confirm that poor eating habits are still widespread among children and that social inequalities have a significant impact on lifestyles and the ability to adopt healthy behaviors. The results highlight the existence of a wide margin for intervention and underline the need for strategies that are not fragmented but community-based. The scientific literature indicates that the most effective initiatives are those based on integrated collaboration between institutions, schools and families. In this context, the Italian school system is already demonstrating significant commitment, but the actions taken need stronger institutional support, more effective involvement of families, and public policies aimed at reducing social inequalities. Only through a coordinated and multisectoral approach will it be possible to promote real and lasting improvements in the health of the younger generation [52,53,54].

Limitations of this study include its cross-sectional design and reliance on self-reported data. Due to the cross-sectional nature of the research, it is not possible to establish a temporal or causal relationship between exposures and outcomes, as both were assessed at the same time. Additionally, the study is limited by the inability to account for all potential variables related to the issue, which may affect the comprehensiveness of the analysis. Some of the variables were self-reported by teachers or parents. The use of BMI as the sole indicator of overweight and obesity could be a limitation because it does not distinguish between body fat and muscle mass. This study also has some strengths. First, the size of the sample evaluated, which is representative at the regional level, allows for the assessment of the importance of the area of residence and differences due to socio-economic conditions. The response rate of parents and the low number of missing data certainly helped to limit selection bias. Finally, standard equipment and methods were used to measure the children by qualified and carefully trained personnel.

## 5. Conclusions

The results presented in this study have highlighted how in Tuscany there is a persistent high level of overweight and obesity in children aged 8–9 years, accompanied by bad eating habits and unhealthy lifestyles. Compared to previous surveys (2008, 2010, 2012, 2014, 2016, 2019), overweight has slightly decreased, while the prevalence of obesity has remained unchanged. The Health Surveillance program “OKkio alla SALUTE” has allowed us to collect useful information to monitor the trend of the variables considered over the years. The scientific literature shows that, to achieve objectives such as promoting healthy eating habits and balanced lifestyles to reduce obesity and overweight in children, it is essential to resort to integrated interventions. These must include the active participation of families, schools and health workers. School is undoubtedly the ideal environment to combat inequalities and promote targeted interventions to reduce social inequalities, enhance food education and encourage physical activity. But only through an intersectoral approach will it be possible to achieve the health standards promoted by the WHO.

## Figures and Tables

**Table 1:** Main characteristics of the participating children.

**Variables**		**N = 1427**
**Child’s sex**		**N (%** **)**
Male		699 (49.4)
Female		716 (50.6)
Missing data		12 (0.8)
**Child’s age**		
8 years		840 (58.9)
9 years		587 (41.1)
**Parents’ educational level**		
Low		206 (15.3)
Medium		603 (44.9)
High		535 (39.8)
Missing data		83 (5.8)
**Area of residence**		
<10,000		136 (9.5)
10,000–50,000		454 (31.8)
>50,000 (no metropolitan area)		399 (28.0)
Metropolitan/Perimetropolitan area		438 (30.7)
**Parents’ citizenship**		
Both Italians		986 (74.9)
One foreign parent		90 (6.8)
Both foreigners		241 (18.3)
Missing data		110 (7.7)
**The family makes ends meet with its earnings**		
Very easily		153 (11.8)
Quite easily		654 (50.5)
With some difficulty		422 (32.6)
With many difficulties		67 (5.2)
Missing data		131 (9.2)

**Table 2:** Outcome variables by sex.

	Boys (N = 699) %	Girls (N = 716) %	Total (N = 1427) * %	*p*-Value
**Fruit/vegetables**				>0.05
Less than once a day	22.9	20.9	22.0	
At least once a day	77.1	79.1	78.0	
Missing data	3.9	2.7	3.2	
**Sugary drinks**				>0.05
At least once a day	18.0	18.8	18.6	
Less than once a day	82.0	81.2	81.4	
Missing data	5.6	4.2	4.9	
**Inactivity ^(1)^**				>0.05
Yes	12.1	11.9	11.9	
No	87.9	88.1	88.1	
Missing data	0.9	0.6	0.7	
**Sedentary lifestyle ^(2)^**				>0.05
Yes	43.2	38.4	40.9	
No	56.8	61.6	59.1	
Missing data	8.3	8.4	8.4	
**Overweight including obesity**				>0.05
Yes	22.6	25.5	24.0	
No	77.4	74.5	76.0	
Missing data	0.6	0.7	1.5	
**Obesity**				>0.05
Yes	7.6	6.5	7.0	
No	92.4	93.5	93.0	
Missing data	0.6	0.7	1.5	

* Twelve cases have no information about sex. ^(1)^ Physical inactivity (not playing outdoors and not doing physical activity) on the day before the interview. ^(2)^ Watching TV or using videogames/tablet/cell phones for more than 2 h on a normal school day.

**Table 3:** Prevalence of outcome variables by socio-economic characteristics.

Socio-Economic Characteristics	Fruit/ Vegetables <Once/Day (%)	*p*-Value	Sugary Drinks ≥Once/Day (%)	*p*-Value	Inactivity ^(1)^ (%)	*p*-Value	Sedentary Lifestyle ^(2)^ (%)	*p*-Value	**Overweight Including Obesity** **(%)**	***p*-Value**	**Obesity** **(%)**	***p*-Value**
**Parents’ educational level**												
Low	24.6	**0.04**	30.6	**<0.001**	9.8	0.18	54.4	**<0.001**	30.8	**0.002**	10.4	**0.002**
Medium	23.9		18.6		13.8		44.8		26.6		8.4	
High	18.2		12.1		10.9		32.4		19.7		4.2	
**Parents’ citizenship**												
Both Italians	21.8	0.99	12.9	**<0.001**	11.0	0.26	38.4	**0.002**	22.9	0.26	6.3	0.24
One foreign parent	22.2		28.4		15.7		47.1		25.8		5.6	
Both foreigners	22.1		34.6		13.7		50.7		27.8		9.3	
**The family makes ends meet with its earnings**												
Very easily	15.7	**0.04**	14.9	0.13	10.5	**<0.001**	22.9	0.81	19.3	**<0.001**	4.7	**0.02**
Quite easily	20.6		17.4		12.5		39.6		20.6		5.4	
With some difficulty	24.0		18.3		11.9		48.2		30.7		10.1	
With many difficulties	30.3		28.1		9.1		49.2		36.4		9.1	
**Area of residence**												
<10,000	20.9	0.84	21.8	0.51	10.3	**0.03**	39.8	0.60	20.3	0.22	6.8	0.63
10,000–50,000	22.9		17.2		13.3		42.0		26.7		7.0	
<50,000 (not metropolitan)	20.6		17.6		14.8		42.7		25.1		8.4	
Metropolitan/perimetropolitan	22.6		20.1		8.4		38.3		21.5		6.0	

^(1)^ Physical inactivity (not playing outdoors and not doing physical activity) on the day before the interview. ^(2)^ Watching TV or using video-games/tablet/computers/cell phones for more than 2 h on a normal school day.

**Table 4:** Mutually adjusted odds ratios for the reported variables—logistic regression.

Variables	Fruit/Vegetables Less Than Once a Day Yes vs. No		Sugary Drinks at Least Once a Day Yes vs. No		Inactivity ^(1)^ Yes vs. No		Sedentary Lifestyle ^(2)^ Yes vs. No		Overweight Including Obesity Yes vs. No		Obesity Yes vs. No	
	OR_adj	95%CI	OR_adj	95%CI	OR_adj	95%CI	OR_adj	95%CI	OR_adj	95%CI	OR_adj	95%CI
**Child’s sex**												
Female	1		1		1		1		1		1	
Male	1.08	0.82–1.43	1.01	0.74–1.38	1.07	0.75–1.52	1.12	0.88–1.42	0.81	0.62–1.06	1.08	0.69–1.71
**Parents’ educational level**												
High	1		1		1		1		1		1	
Medium	1.24	0.91–1.70	**1.55**	**1.07–** **2.24**	1.28	0.87–1.88	**1.50**	**1.15–1.96**	1.29	0.95–1.74	1.64	0.95–2.84
Low	1.42	0.90–2.23	**1.97**	**1.22–3.18**	0.69	0.36–1.32	**1.99**	**1.33–2.97**	1.42	0.92–2.18	1.71	0.83–3.51
**Area of residence**												
<10,000	1		1		1		1		1		1	
10,000–50,000	1.22	0.73–2.05	0.91	0.52–1.58	1.37	0.70–2.66	1.12	0.71–1.75	1.57	0.92–2.67	1.41	0.56–3.53
>50,000 (no metropolitan)	1.01	0.59–1.72	0.95	0.54–1.68	1.45	0.74–2.87	1.15	0.73–1.83	1.65	0.96–2.84	1.88	0.75–4.72
metropolitan/perimetropolitan	1.17	0.69–1.97	0.83	0.47–1.45	0.81	0.40–1.63	0.91	0.58–1.44	1.23	0.71–2.10	0.93	0.36–2.42
**Parents’ citizenship**												
Both Italians	1		1		1		1		1		1	
One foreign parent	0.98	0.57–1.69	**2.73**	**1.60–** **4.64**	1.37	0.71–2.64	1.32	0.82–2.11	1.05	0.63–1.78	0.87	0.33–2.26
Both foreigners	0.83	0.56–1.22	**2.** **89**	**1.98–4.22**	1.41	0.88–2.27	1.28	0.91–1.81	1.20	0.83–1.71	1.56	0.89–2.73
**The family makes ends meet**												
**with its earnings**												
Very easily	1		1		1		1		1		1	
Quite easily	1.44	0.87–2.39	0.94	0.54–1.62	1.36	0.73–2.55	**1.97**	**1.26–** **3.08**	1.08	0.67–1.76	1.06	0.43–2.61
With some difficulty	1.59	0.93–2.73	0.83	0.46–1.49	1.21	0.62–2.36	**2.53**	**1.58–** **4.05**	**1.** **81**	**1.09–2.98**	1.98	0.80–4.82
With many difficulties	**2.35**	**1.12–** **4.92**	1.36	0.61–3.06	0.86	0.29–2.59	**3.30**	**1.66–** **6.56**	2.01	0.98–4.10	1.43	0.38–5.40

^(1)^ Physical inactivity (not playing outdoors and not doing physical activity) on the day before the interview. ^(2)^ Watching TV or using videogames/tablet/computers/cell phones for more than 2 h on a normal school day.

## Data Availability

OKkio alla SALUTE data and questionnaires can be accessed via a re- quest to the Principal Investigator, Prof. Giacomo Lazzeri (giacomo.lazzeri@unisi.it). For further information, please see https://www.epicentro.iss.it/okkioallasalute/ (accessed on 4 July 2025).

## Abbreviations

The following abbreviations are used in this manuscript:

BMI	Body Mass Index
Cis	Confidences intervals
COSI	Childhood Obesity Surveillance Initiative
ISS	Italian National Institute of Health
LHU	Local Health Unit
ORs	Adjusted odds ratios
WHO	World Health Organization
WOF-IOFT	World Obesity Federation—International Obesity Task Force
